# Sleep quality and nocturnal pain in patients with elbow disorders: a prospective multicenter study

**DOI:** 10.1186/s10195-026-00950-6

**Published:** 2026-07-15

**Authors:** Marco M. Schneider, Rainer Nietschke, Fillipo Migliorini, Christian Schoch, Klaus Burkhart, Boris Hollinger, Michael Kimmeyer, Lars Lehmann, Alexander Zimmerer

**Affiliations:** 1 Fakultät für Gesundheit, University of Witten / Herdecke, Alfred-Herrhausen-Str. 50, 58455 Witten, Germany; 2 Praxisklinik Orthopädie Aachen (PKO), Aachen, Germany; 3 Gelenkquartier, Orthopaedie and Unfallchirurgie, Karlsruhe-Durlach, Germany; 4 Department of Trauma and Reconstructive Surgery, Martin-Luther University Halle-Wittenberg, Halle (Saale), Germany; 5 Department of Life Sciences, Health, and Health Professions, Link Campus University, Rome, Italy; 6 Department of Orthopedics and Trauma Surgery, Academic Hospital of Bolzano (SABES-ASDAA), Bolzanom, Italy; 7 St. Vinzenz Klinik Pfronten, Pfronten, Germany; 8 Sportorthopaedie, Diakonie Klinikum Stuttgart, Stuttgart, Germany; 9 Orthopaedische Klinik Markgroeningen, Abteilung Sportorthopaedie, Markgroeningen, Germany; 10 Department of Orthopaedic and Trauma Surgery, University Medical Centre Mannheim, Medical Faculty Mannheim, University of Heidelberg, Mannheim, Germany; 11 Department of Traumatology, Hand Surgery and Sports Medicine, ViDia Clinics Karlsruhe, Karlsruhe, Germany; 12 Department of Orthopedics and Orthopedic Surgery, University Medicine Greifswald, Greifswald, Germany; 13 Faculty of Medicine, Center of Orthopedic and Trauma Surgery, University Hospital Cologne, University of Cologne, Cologne, Germany; 14 Sektion für Gelenk- und Extremitätenchirurgie, Uniklinik RWTH Aachen, Aachen, Germany

**Keywords:** Sleep Quality, Nocturnal pain, elbow, PSQI, night

## Abstract

**Background:**

Mental health disorders have become a growing global concern. Sleep quality is known to have a crucial impact on mental well-being. Poor sleep contributes to increasing economic costs owing to reduced productivity as well as the development of mental disorders. Conversely, anxiety and depression often lead to sleep disturbances. Pain is a key factor affecting sleep quality. Studies have shown that musculoskeletal disorders, especially in the shoulder, may cause sleep disturbances. Research on the relationship between elbow pain and sleep quality remains limited. This study aims to evaluate the impact of common elbow pathologies on sleep quality.

**Methods:**

This prospective multicenter cohort study used self-reported outcome measures in patients with elbow disorders, including the Pittsburgh Sleep Quality Index (PSQI), to assess sleep quality. The PSQI was compared with other outcome measures.

**Results:**

A total of 664 patients (284 female, 380 male) were included, of whom 81% reported nocturnal pain. The mean age was 47.9 ± 14.2 years, body mass index (BMI) 26.9 ± 6.7 kg/m^2^ and symptom duration 15.8 ± 7.3 months. The most common diagnoses were lateral epicondylar pain (66%), medial epicondylar pain, arthritis, ulnar nerve entrapment and biceps/triceps tendinopathies. The overall PSQI was 7.3 ± 3.8, disabilities of arm, shoulder and hand questionnaire (DASH) 35.0 ± 19.8, sports/performing art module of disabilities of arm, shoulder and hand questionnaire (spa-DASH) 16.2 ± 13.8, work module of disabilities of arm, shoulder and hand questionnaire (w-DASH) 29.2 ± 15.8, single assessment numeric evaluation elbow (SANE-E) 54.9 ± 25.4 and numeric rate scale (NRS) 3.1 ± 2.7. No significant differences in other scores were observed between pathology subgroups. Correlation analysis showed a moderate association between PSQI and DASH score (*r* = −0.372, *p* < 0.001), as well as between PSQI and NRS for pain (*r* = 0.255, *p* = 0.002), while other variables showed no significant correlations.

**Conclusions:**

Nocturnal pain and sleep disturbances are highly prevalent in patients with elbow disorders. Evaluating and addressing sleep quality may improve functional outcomes and overall well-being. Future research should investigate how different treatment modalities affect pain, function and sleep quality.

## Introduction

Mental health disorders have become a significant global health concern, with a rising incidence in recent years owing to the COVID pandemic, among other reasons [1, 2]. According to the World Health Organisation (WHO), depression and anxiety disorders affect millions of people worldwide [3]. Researchers found numerous factors contributing to the rising burden of mental health disorders, including predisposition, lifestyle and environmental influences. One crucial but often underestimated consideration influencing mental health is sleep and sleep quality. Sleep plays a vital role in cognitive function, emotional regulation and overall mental well-being [4–7]. Poor sleep can contribute to the emergence of mental disorders and influence reduced work hours as well as mortality [8–10]. Conversely, patients with anxiety and depression often suffer from sleep disturbances, which have been proven to be responsible for high economic costs [11, 12]. Therefore, recognising factors that might negatively impact sleep is essential to reduce mental health problems and improve quality of life.

Pain is a key contributor to impaired sleep quality and can lead to difficulty falling asleep, frequent awakenings and reduced sleep quality [13]. Musculoskeletal pain has been associated with sleep disturbances in several studies [14–17], the shoulder being one of the most affected joints, especially in patients suffering from rotator cuff pain, osteoarthritis and frozen shoulder [18–22]. Depending on the underlying pathology, rotator cuff repair or shoulder arthroplasty can reduce night pain and improve sleep quality [23–25]. These findings underscore the importance of addressing musculoskeletal pain, not only for physical recovery but also to enable better sleep and elevate psychological well-being. Research on the association between elbow pain and sleep disturbance is limited. Given the growing awareness of the importance of sleep quality for global health, investigating whether similar patterns exist among patients with elbow pathologies seems useful. This study aims to evaluate the association between common elbow pathologies and sleep quality by examining the prevalence of nocturnal pain and comparing sleep quality across diagnostic subgroups. We hypothesised that patients with higher pain intensity and greater functional impairment would demonstrate worse sleep quality as measured by the PSQI. The primary objective of this study was to assess sleep quality in patients with common elbow pathologies using the PSQI and to evaluate its association with pain intensity and functional impairment. Secondary objectives included comparing sleep quality across diagnostic subgroups and identifying patient-related factors associated with impaired sleep quality. The findings are expected to provide physicians with a deeper understanding of the burden experienced by patients with elbow disorders and to emphasise the impact of sleep disturbance when considering surgical interventions.

## Materials and methods

This prospective, multicenter cohort study was initiated after approval from the local institutional review board.

### Patient selection

**Fig. 1:**
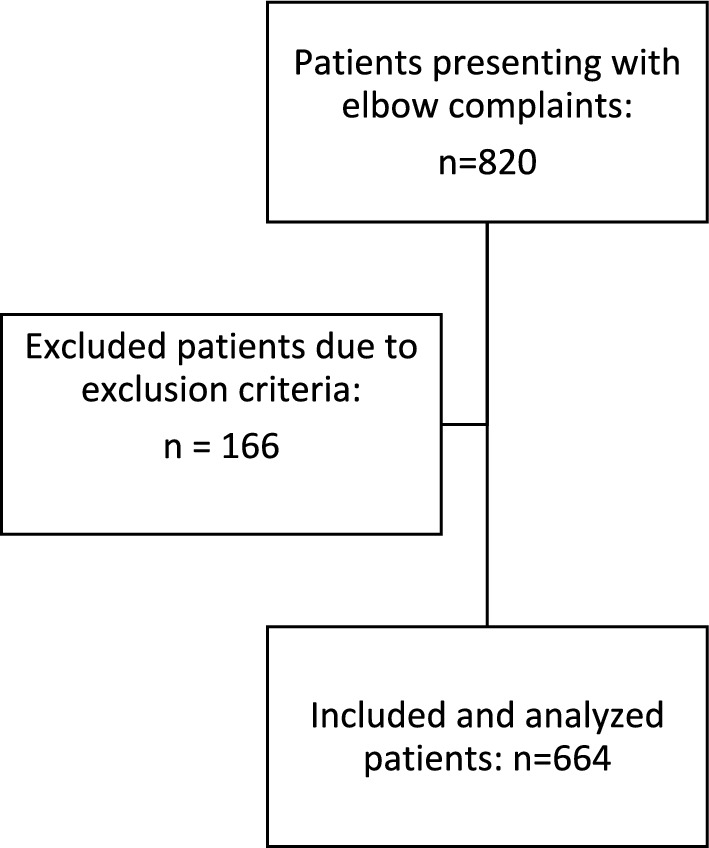
Flow chart of patient inclusion

This study was conducted between December 2020 and May 2024, including 664 consecutive patients enrolled across five different orthopaedic surgery departments, each contributing their own patient cohort from individuals who presented with complaints around the elbow joint and met the inclusion criteria (Fig. 1). All patients aged 18 years or older at the time of the first consultation were included in the study. Patients were excluded if they had undergone prior elbow surgery (*n* = 44), experienced a recent fracture (within the past 2 years) (*n* = 29), had a documented history of mood disorders (*n* = 22), current substance abuse issues (*n* = 11), a diagnosed sleep disorder (*n* = 17), other joint pathologies contributing to sleep disturbances (*n* = 38) or did not provide consent to participate (*n* = 5). Patients with a suspected diagnosis of posterolateral rotatory instability or medial elbow instability were also excluded, as the reliable assessment of these conditions is highly examiner-dependent and could not be standardized across the five participating centres. The enrolled patients were asked to complete a questionnaire assessing their current medical history, with details regarding the presence of mental health disorders, elbow-related symptoms and the impact of symptoms on activities of daily life (ADL) and sleep quality. Data collection was standardised by using self-reported outcome instruments, including the Pittsburgh sleep quality index (PSQI) [26], disabilities of arm, shoulder and hand questionnaire (DASH) [27–29], with optional inclusion of the work and sports/performing art module and single assessment numeric evaluation elbow (SANE-E) forms. Pain was assessed using the numeric rate scale (NRS). Information was gathered before the consultation with the orthopaedic surgeon. Additionally, the patient completed a medical history form that included details of the medical problem and current and prior comorbidities. The Pittsburgh sleep quality index (PSQI) is a questionnaire for self-assessment of sleep quality over the past 4 weeks [26]. The test consists of 19 self-assessment questions and five external assessment questions. It evaluates different sleep-related parameters, including the frequency of sleep-disrupting events, subjective sleep quality, usual sleep times, sleep duration, sleep latency and daytime sleepiness. A total of 18 items from these categories are used for quantitative evaluation. The test is structured into seven categories, each assigned a value ranging from 0 to 3. The sum of all seven component scores yields the total score (ranging from 0 to 21), with higher scores indicating poorer sleep quality. The PSQI has been validated in several languages and demonstrates sensitivity and specificity of over 80% for distinguishing poor sleepers from good sleepers using a global score cut-off of > 5 [26, 30, 31]. The stability of the test is satisfactory with good test reliability; a Cronbach alpha for the component value sleep disturbance was 0.77 [30].

### Functional restrictions of the elbow owing to diseases (DASH, SANE)

The most commonly used questionnaire for assessing functional impairment due to upper extremity injuries or degenerative diseases is the DASH score. This instrument evaluates the patient’s perception of upper extremity functional limitations. It consists of 30 items covering seven different domains of daily life: family and domestic obligations, recreation, social activities, work, sexual activity, self-care and essential activities. Furthermore, participants completed two supplementary modules: the sports/performing arts module (spa-DASH) and the work module (w-DASH), each consisting of four additional questions. To determine the patient’s SANE-E score, patients were asked: “How would you rate your elbow today as a percentage of normal (scale of 0–100%, with 100% being normal)?” The criterion validity of the SANE-E was assessed by comparing it with other validated outcome measures, with a correlation coefficient (*r*) of 0.62 with the ASES-E scale of 0.62 [32].

### Individual pain rating

Finally, NRS was gathered to assess the patient’s pain intensity. Additionally, patients were asked whether they experienced symptoms in other joints besides the elbow and whether these symptoms affected their sleep quality. In cases with significant sleep disturbance primarily caused by other joint pathologies, patients (*n* = 38) were excluded from the study.

### Statistics

Statistical analyses were performed in accordance with the predefined primary and secondary study objectives. Descriptive statistics were used to analyse patient demographics and prevalence data. Differences between groups were assessed using a one-way analysis of variance. Differences between groups were assessed using a one-way analysis of variance. When data did not meet the Shapiro–Wilk normality test requirements or were non-parametric, a Kruskal–Wallis analysis was performed. If a patient was affected bilaterally, only the more painful or more functionally impaired limb was included in the analysis. To identify preoperative patient characteristics associated with poor sleep quality (e.g., higher PSQI scores), a Spearman correlation analysis was performed. The variables included in the correlation analysis were age, sex, body mass index (BMI) and symptom duration (months). Based on an expected correlation coefficient of 0.20, a type I error probability of 0.05, and a power of 0.80, a sample size of 216 participants was required. All statistical analyses were performed using XLStat (Addinsoft Inc, NY, USA).

## Results

A total of 664 patients met the inclusion criteria. The study included 284 female and 380 male patients, of whom 81% reported nocturnal pain. The right elbow was affected in 484 (72%) patients, while the left elbow was painful in 188 (28%). The dominant elbow was involved in 547 (75%) patients. The mean age of the cohort was 47.9 ± 14.2 years (range 18–85 years), and the average BMI was 26.9 ± 6.7 kg/m^2^ (range 18.3–32.5 kg/m^2^). The mean symptom duration since onset was 15.8 ± 7.3 months (range 2–36 months). Among the 664 patients, the five most common elbow pathologies were lateral epicondylar pain, medial epicondylar pain, arthritis, ulnar nerve entrapment and tendinopathies of biceps or triceps. Less common diagnoses, such as olecranon bursitis and elbow stiffness, were pooled as “other” in a sixth group. Lateral epicondylar pain was the most prevalent diagnosis, affecting 66% of patients. The descriptive data for the different pathologies and their subgroups are shown in Table 1. Among all groups, the arthritis subgroup was the only one to show a statistically significant difference in age and symptom duration compared with the other subgroups. The overall PSQI was 7.3 ± 3.8 (1–20), the overall DASH score was 35.0 ± 19.8 (1–83), the overall spa-DASH score was 16.2 ± 13.8 (1–100), the overall w-DASH score was 29.2 ± 15.8 (1–100), the SANE-E score was 54.9 ± 25.4 (1–100) and the NRS for pain was 3.1 ± 2.7 (0–10), respectively. The scores for the pathology subgroups are shown in Table 2. The arthritis group had a slightly lower PSQI score than the other groups. However, this difference did not reach significance (*p* > 0.05). For the other scores, no significant differences were observed between the groups. Correlation analysis revealed moderate correlations between the DASH score (*r* = −0.372; *p* < 0.001), NRS for pain (*r* = 0.255; *p* = 0.002) and the reported PSQI. No significant correlation was found for the remaining variables analysed.

**Table 1:** Study population demographic data by pathology classification

Characteristic	Lateral epicondylar pain (*n* = 444)	Medial epicondylar pain (*n* = 60)	Arthritis (*n* = 38)	Ulnar nerve entrapment (*n* = 34)	Tendon (*n* = 34)	Others (*n* = 54)
Age, years	49.4 ± 10.2 (31–80)	48.5 ± 12.5 (28–73)	63.7 ± 12.8 (46–85)*	44.8 ± 15.5 (18–75)	46.0 ± 13.5 (23–83)	47.5 ± 12.7 (23–76)
Side of involvement (right/left)	196/248	50/10	22/16	18/16	18/16	28/26
Gender (male/female)	258/186	32/28	32/6	28/6	34/0	30/24
BMI, kg/m^2^	26.9 ± 6.7 (18.3–32.5)	26.8 ± 6.5 (19.1–32.2)	26.7 ± 4.5 (21.3–31.4)	25.5 ± 4.5 (18.3–30.4)	26.3 ± 5.5 (19.6–27.6)	26.9 ± 6.7 (19.3–31.5)
Time since onset of symptoms, months	11.0 ± 8.3 (2–36)	12.2 ± 6.5 (2–33)	19.7 ± 9.5 (6–36)*	13.5 ± 8.5 (3–36)	11.8 ± 7.5 (6.5–35)	15.8 7.3 (2–36)

Values are shown as *n* (%) or mean ± standard deviation (range). BMI, body mass index

*p < 0.001

**Table 2:** Subject population pain, sleep and function scores by pathology classification

Characteristic	Lateral epicondylar pain (*n* = 444)	Medial epicondylar pain (*n* = 60)	Arthritis (*n* = 38)	Ulnar nerve entrapment (*n* = 34)	Tendon (*n* = 34)	Others (*n* = 54)
PSQI	7.6 ± 4.1 (2–20)	6.6 ± 3.3 (2–11)	5.9 ± 3.8 (1–10)	7.6 ± 3.8 (4–14)	7.2 ± 3.5 (3–11)	7.5 ± 3.9 (1–15)
DASH	34.4 ± 18.1 (1–80)	26.8 ± 18.0 (3–75)	35.7 ± 19.0 (4–55)	29.2 ± 18.7 (6–79)	35.7 ± 19.0 (4–55)	33.7 ± 1.85 (2–100)
spa-DASH	13.3 ± 9.1 (2–93.8)	17.3 ± 11.1 (2–100)	18.3 ± 12.3 (2–90)	18.3 ± 12.3 (2–90)	14.3 ± 9.3 (2–93.8)	18.3 ± 12.3 (2–90)
w-DASH	36.0 ± 14.1 (2–93.8)	26.3 ± 9.1 (1–87.5)	30.3 ± 11.1 (2–91)	30.3 ± 11.1 (2–91)	37.0 ± 14.7 (2–100)	30.3 ± 11.1 (2–91)
SANE-E	55.0 ± 25.7 (0–100)	59.6 ± 25.9 (15–95)	72.5 ± 13.5 (55–75)	38.5 ± 13.8 (0–55)	52.5 ± 23.5 (10–65)	72.5 ± 13.5 (55–75)
NRS	3.3 ± 2.6 (1–9)	2.6 ± 2.3 (0–8)	3.0 ± 1.2 (2–6)	3.0 ± 2.2 (0–6)	3.6 ± 2.8 (2–10)	3.2 ± 1.2 (2–6)

Values are shown as mean ± standard deviation (range)

DASH, disabilities of arm, shoulder and hand questionnaire; NRS, numeric rate scale; PSQI, Philadelphia sleep quality index; SANE-E, single assessment numeric evaluation elbow; spa-DASH, sports/performing art module of disabilities of arm, shoulder and hand questionnaire; w-Dash, work module of disabilities of arm, shoulder and hand questionnaire

## Discussion

This study evaluates nocturnal pain and sleep quality in a large patient population with elbow pathologies. It demonstrates that nocturnal pain and sleep disturbance are prevalent in patients with elbow pathologies. Overall, 81% of patients reported elbow pain at night. The impact of elbow-related discomfort on sleep quality is substantial, although the prevalence is slightly lower compared with studies on shoulder pathologies, in which patients report night pain up to 91% [22]. However, it is still significant and comparable to pain-related sleep disturbance seen in spinal disorders and hip pathologies, with up to 74% and up to 100%, respectively [14, 16, 33, 34]. This study evaluated nocturnal pain and sleep quality in a large patient population with elbow pathologies.

In our study, the mean Pittsburgh sleep quality index (PSQI) of 7.3 ± 3.8 (1–20) across all elbow pathologies indicates poor sleep quality, as scores above the threshold of 5 are considered significant [22]. A PSQI score greater than 5 was observed in 66% of patients with lateral epicondylar pain (LEP), 70% of patients with ulnar nerve entrapment (UNE), 70% of patients with arthritis, 60% of patients with medial epicondylitis (ME) and 70% of patients in the ‘others’ category. This finding aligns with studies on other joints, such as the shoulder. Several studies have reported poor sleep quality as a consequence of pain in patients with osteoarthritis [22, 23], frozen shoulder [19, 22, 35] or rotator cuff tears. Notably, PSQI scores in rotator cuff studies (8.59 and 11.6, respectively) were higher than those observed in our cohort [18, 20, 21, 25].

Little is known about the effect of elbow pathologies on sleep quality. Giöstad et al. [36, 37] examined patients with ulnar nerve entrapment (UNE) and found that 68% reported pre-operative sleeping problems. Moreover, the authors identified pre-operative sleep disturbances as a risk factor for chronic pain after surgery, as only 58% of patients experienced improvement after surgery. In our study, 70% of patients with UNE complained about nocturnal pain. Given that UNE can cause both pain and sensory discomfort (e.g., paresthesia and numbness), these factors may contribute equally to sleep impairment. Regardless of the underlying cause, the findings highlight the importance of assessing sleep quality in patients with UNE during initial and follow-up consultations.

In our study, 66% of patients with lateral epicondylar pain (LEP) described nocturnal pain. This aligns with the findings of Bateman et al. [38], who conducted a qualitative study of patients with lateral epicondylitis (LE). Although not providing specific prevalences, several participants complained about a significant impact on everyday life owing to severe pain at night and, hence, sleep disturbance. Another study of the same group attempted to optimise physiotherapy for patients with LE but did not assess nocturnal pain as a baseline characteristic or outcome measure [39]. Reyhan et al. [40] investigated the effects of Mulligan’s mobilisation with movement in patients with LE and recorded baseline night-time pain on a visual analogue scale (VAS) of 2.20 ± 1.40 in group one and 2.85 ± 0.75 in group two. They found a significant reduction in pain after 3 months of treatment but did not analyse the impact of pain on sleep disturbances [40]. Radwan et al. [41] reported slightly higher nocturnal pain levels in patients with LE, with a VAS score of 30 (out of 100). Their findings are consistent with ours, in which the NRS in patients with LE ranged from 1 to 9 (3.3 ± 2.6) [41].

A key finding in our investigation was the moderate correlation between functional impairment and sleep disturbance. The DASH score correlated negatively with the PSQI (*r* = −0.372; *p* < 0.001), indicating that patients with worse functional scores reported poorer sleep quality. Similarly, NRS and PSQI correlated positively (*r* = 0.255, *p* = 0.002), suggesting that higher pain levels were associated with more sleeping problems. Similar to our findings, Zhang et al. [42], Horneff et al. [43] and Cho et al. [44] found higher PSQI scores in patients with elevated VAS pain scores in shoulder patients. We did not find any other correlation between PSQI and other parameters. The lack of correlation of the PSQI with age, BMI or symptom duration suggests that sleep disorders in patients with elbow pathologies are mainly caused by pain.

The reasons for elevated PSQI scores and night-time pain in patients with elbow disorders remain unclear. Tendinopathy in patients with LE and medial epicondylitis (ME) leads to the release of pro-inflammatory and pain-inducing cytokines, similar to injuries or tendinopathies of the rotator cuff [45]. Haack et al. [46] have several suggestions for the potential mechanisms explaining the correlation between sleep deficiency and pain, including ‘opioid, monoaminergic, orexinergic, immune, melatonin and endocannabinoid systems; the hypothalamus–pituitary–adrenal axis; and adenosine and nitric oxide signaling.’ In particular, the influence of melatonin has been the focus of numerous studies. Ha et al. [47] described a potential association between melatonin levels and night-time pain in patients with rotator cuff tears and frozen shoulder. In a study by Perez et al. [48], melatonin intake following rotator cuff repair led to an improvement in PSQI. However, whether the same effect occurs in elbow pathologies remains unclear.

The findings of our study emphasise the importance of assessing sleep quality in patients with elbow pathologies. With the lack of studies investigating sleep disturbances and nocturnal pain in patients with elbow disorders, these aspects seem to be an overlooked aspect. Poor sleep quality has a huge influence on daily life and causes enormous economic costs [9, 12]. Based on our results with a large sample size, physicians, especially elbow surgeons, should consider questions about night-time pain and sleep disturbances in patients with elbow pain, particularly in cases of UNE and LE, which have the highest prevalence of symptoms.

The present study has limitations. The Pittsburgh sleep quality index is a subjective measure of sleep quality based on patients’ self-reports. The use of objective measurement methods, such as actigraphy, and additional scales, such as the Epworth sleepiness scale, could have provided a more accurate assessment of sleep patterns. Another limitation is that data collection was based on self-reported questionnaires. This can lead to biases due to patients’ subjective perceptions, although patients with known mental health disorders were excluded. Furthermore, while patients with instability diagnoses were excluded owing to the examiner-dependent nature of their assessment in a multicentre setting, we cannot entirely rule out that isolated cases with an underlying diagnosis of instability may have been inadvertently included in the lateral or medial epicondylar pain cohort. The inclusion of patients with ulnar nerve entrapment may be considered a limitation, as symptoms often extend beyond the elbow; however, this inclusion was justified by the study’s focus on sleep disturbance.

## Conclusions

Our study illustrates that sleep disturbances are highly prevalent among patients with elbow pathologies, with 81% of the patients reporting nocturnal pain and reduced sleep quality measured by elevated PSQI scores. By assessing and addressing sleep quality in the management of elbow disorders, clinicians might improve functional outcomes and general well-being. Future studies should explore the effects of different therapeutic modalities, such as physiotherapy, injections and surgery, on pain and function, and their correlation with sleep quality.

## Data Availability

The data supporting the findings of this study are available from the corresponding author upon reasonable request.

## Declarations

### Use of artificial intelligence (AI)

Language editing was performed using Grammarly (Superhuman Platform Inc., San Francisco, CA, USA).
